# The ecosystem of human capital in care homes

**DOI:** 10.3389/fpubh.2024.1298833

**Published:** 2024-03-04

**Authors:** Anamarija Kejžar, Katri Maria Turunen

**Affiliations:** ^1^Chair of Long-Term Care, Faculty of Social Work, University of Ljubljana & MRRC UL SI-AHA, Ljubljana, Slovenia; ^2^Seinäjoki University of Applied Sciences, Seinäjoki, Finland

**Keywords:** tacit knowledge, care home, intellectual capital, knowledge transfer, experts from experience

## Abstract

**Introduction:**

This qualitative study addresses the essential yet often overlooked experiences of knowledge transfer within care homes (CH). Conducted in a Slovenian CH in 2020 and 2023, participants, including CH management, staff, and residents with their relatives, shared perceptions of knowledge transfer at various levels. The study aims to explore barriers and facilitators for knowledge transfer crucial for creating new knowledge, services, and enhancing care quality for older individuals.

**Methods:**

Structured focus group interviews were conducted, and data were collected within the CH. The participants’ insights into knowledge transfer were probed, covering various dimensions such as between individuals, groups, organizations, and the community. Transcriptions of recorded interviews were analyzed using content analysis.

**Results:**

Knowledge transfer within the CH was facilitated through continuous training, diverse communication channels, and mentoring. Collaboration with relatives improved understanding of resident preferences, habits, and overall enhanced the quality of care. This collaborative effort allowed mutual learning and knowledge transfer from the CH to the broader community. Despite potential benefits, there is an underutilization of information and communication technology, e-care, and untapped potential for partnerships, partly due to the scarcity of care. Barriers were identified in the form of stereotypical attitudes towards aging and care, further reinforced by negative news coverage on older people’s care.

**Conclusion:**

The multidimensional nature of knowledge in CH centers on resident well-being, emphasizing three key aspects of knowledge transfer: between staff and residents, staff and residents’ families, and between the CH and the community. In the context of age management, creating opportunities for knowledge transfer is crucial, emphasizing a transition from traditional institutional care to an approach prioritizing knowledge about quality care. and involving experts from experiences in care process.

## 1 Introduction

According to the Community of Social Institutions of Slovenia (1), 103 public and private CH offer 19,403 places for older adults. The number of people in Slovenia aged 65 and over is 453,708, which means that this capacity is sufficient for 4.35 percent of the older adults population. Depending on demand, it is necessary to wait for admission to some CH in Slovenia for several years. The phenomenon is not limited to Slovenia but broadly affects several European countries where the number of older adults in need of care is rising, and resources are insufficient to meet the demand (2). Over the last several decades, there has been a shift in the focus of quality of services and quality of living in care homes (CH). Although physical care used to be the most prominent focus, recently, there has been a shift toward a holistic view of the person and person-centered care, which requires seeing the whole person and their physical, psychological, spiritual, and social dimensions. There has been a change from more passive care to active care, with meaningful activities highlighted as important in long-term care (3). There have been multiple high-quality trials and systematic reviews that provide evidence for good practice in long-term residential institutions for older adults, referred to in many countries as nursing homes, also known as long-term CH, homes for the aged, rest homes, and residential aged care facilities (4–6).

This paper describes the existing literature on the knowledge ecosystem in CH. Specifically, this paper highlights three well-polarized clusters: knowledge of employees (specially carrers and health professionals), knowledge of residents, and knowledge of relatives. The CH can be seen as a gerontological center in the local community. In the current study, one CH was used as the site for an observational study design to look at social innovative practices and knowledge transfer within CH and from CH to the community. Specifically, factors facilitating and hindering the transfer of knowledge were investigated. The interviews were conducted in 2020 and 1 to allow for pre-and post-Covid-19 research. In CH, the transfer of knowledge and information about residents and collaboration among staff, between staff and residents, and between staff and family members is vital for the implementation of quality of care and the well-being of residents. Together, they form an ecosystem of the intellectual capital of CH. From the point of view of intellectual capital, the paper discusses different forms of tacit knowledge in CH, various methods of knowledge transfer and possible obstacles. The focus of research was in identifying the facilitators and barriers to implementing knowledge transfer in a CH and in relationship between CH and community. Intellectual capital can be viewed as an ecosystem of knowledge within an organization. This concept emphasizes the interaction and integration of different components of intellectual capital, such as human capital, structural capital, and relational capital, to create a dynamic and value-creating environment. The idea that intellectual capital forms an ecosystem is discussed by Edvinsson and Malone (7), Sveiby (8), and Nonaka and Takeuchi (9). Edvinsson and Malone (7) examine how different forms of intellectual capital work together to create value within an organization. They describe intellectual capital as a living ecosystem of knowledge. Human capital, social capital, and structural capital are intertwined, and their synergy often leads to innovation and competitive advantage (8). Human capital refers to the knowledge, skills, and experience formed by investment in education and training (8, 10). It includes explicit and implicit human capital (11). Explicit human capital refers to the external components that make up the value of human capital and can be measured using standard methods, such as education and service length (11). Implicit human capital refers to employees’ knowledge, experience, creativity, and value systems (11). Implicit human capital is more original and fundamental than explicit human capital, and it is the wellspring of innovation performance and the cornerstone of all explicit knowledge (11). Subramaniam and Youndt (12) point out that human capital and social capital are two primary aspects of intellectual capital, and there is a significant synergistic effect between them (13, 14). The term social capital initially appeared in community studies to highlight the central importance – for the survival and functioning of city neighborhoods – of networks of strong, crosscutting personal relationships developed over time that provide the basis for trust, cooperation, and collective action in such communities (15). Social capital is one of the components of intellectual capital that refers to the value derived from an organization’s external relationships, networks, and interactions with its stakeholders, such as customers, suppliers, partners, and the community. It encompasses the goodwill, trust, reputation, and social capital that an organization builds through its connections with external entities (16, 17). Rich social capital can effectively improve employee task and contextual performance (18).

Structural capital refers to an organization’s non-human assets embodied and stored in information systems, databases, programs (19), production processes, information technologies (20), information systems, workflows, know-how (21), innovations, business processes (19, 22), organizational capabilities, culture, and intellectual property (23). This capital is owned by an organization and remains in that organization when its employees or members of the organization are no longer part of it (24). Managers should be aware of and understand the concept of intellectual capital within an organization for five reasons:

1. Strategic decision-making: Intellectual capital plays a critical role in strategic decision-making. Managers need to know the intellectual capital of their companies to make informed decisions about resource allocation, innovation, and market positioning. Understanding the strengths and weaknesses of intellectual capital can guide the development of effective strategies (7).
2. Competitive advantage: Intellectual capital often serves as a source of sustainable competitive advantage. Managers who know the intellectual capital of a firm can use it to outperform their competitors and create unique products or services and social innovations.
3. Resource allocation: Knowing a company’s intellectual capital helps managers allocate their resources more efficiently. For example, they can invest in training and development programs to improve human capital or allocate resources to protect and develop knowledge assets (8).
4. Risk management: Understanding intellectual capital enables managers to identify and mitigate the risks associated with knowledge loss. Employee turnover, retirement, or sudden departure can lead to the loss of important knowledge assets. Managers who are aware of these risks can implement strategies to capture and transfer knowledge (25).
5. Measurement and reporting: Intellectual capital can be measured and reported, which helps managers communicate the intangible value of their firms to stakeholders, including shareholders. This transparency can enhance trust.

The knowledge ecosystem highlights the significance of knowledge sharing and collaboration within an organization. When employees share their expertise and collaborate effectively, an organization’s overall intellectual capital is strengthened (9, 26). As intellectual capital within an ecosystem flows and evolves over time, managers should facilitate the flow of knowledge among individuals, teams, and departments to maximize the potential of their organization’s intellectual capital. The ecosystem perspective emphasizes that intellectual capital, when managed effectively, leads to value creation. Companies that nurture their intellectual capital ecosystem are more likely to adapt to changing circumstances, innovate, and achieve sustainable success.

In summary, human capital as part of intellectual capital can be viewed as an ecosystem of knowledge within organizations. This perspective highlights the interconnectedness of various knowledge resources and their role in creating value, fostering innovation, and facilitating adaptability.

### 1.1 Knowledge in care homes

Explicit knowledge typically refers to knowledge that has been expressed in words and numbers. Such knowledge can be shared formally and systematically in the form of, for example, data, drawings, audio and video tapes, and computer programs (27). Explicit knowledge in CH represents documentation and records about:

1. residents – their medical documentation, biographic stories, and care plans),
2. documented staff meetings and shift handovers, feedback mechanisms, CH standards and procedures, CH quality systems, books and training programs, CH information technology, and data security and privacy, and
3. different stakeholders.

In contrast, tacit knowledge includes insights, intuitions, and hunches. This knowledge is difficult to express and formalize and therefore difficult to share. Despite written protocols, the knowledge contained in the implementation of the service is intertwined with the attitude of an individual caregiver to a resident. The key to quality care is often in undocumented information, including intuition, empathy, and experience, which enables us to make the right decisions, as Gamble and Blackwell (28) describe. Tacit knowledge is of great importance in delivering quality services because it is an essential part of creating relationships with residents, listening to them, and delivering person centered care. Tacit knowledge can be the most important element in promoting the well-being of CH residents and maintaining the meaning in residents’ lives. It is internalized in staff attitudes toward residents, in creating livability, in respecting residents’ wants and needs, and in the way staff feed, care for, and talk to residents. As it creates an important part of human capital that an organization does not own, it is strategically important to enable and foster knowledge transfer in CH. But one important, often neglected type of knowledge, is knowledge of users – experts by experience. Innovation research and practice is increasingly focusing on the experience of service users. Through methods such as ethnography, service design and co-production, there is a growing realization that it is not enough to talk to a few people about their experiences, but that people really need to be involved in the design and delivery of services if they are to work – which affects the quality of both social and health care services in CH (29–31).

### 1.2 Knowledge transfer in care homes

Knowledge transfer in CH is critical to ensure that important information, skills, and expertise are effectively shared among staff and passed from experienced caregivers to new staff. Effective knowledge transfer can lead to improved resident care, increased staff competency, and a conducive work environment. Knowledge transfer can be consistently promoted in a variety of ways to capture tacit knowledge, share it from individual tacit knowledge to organizational level, such as through the: (1) physical level – in face-to-face training; mentoring; meetings, counselling; (2) virtual level – through email, teleconferencing, videos, tutorials, clouds, intranet; (3) mental level – as shared experiences, ideas, values, beliefs; and (4) relationship level – people are sharing common goals, expectations, needs, strengths and weaknesses.

A consistent and important method of knowledge transfer are regular staff meetings at which staff discuss best practices, share lessons learned, and address challenges residents face. Effective knowledge transfer helps new staff learn from experienced staff, minimizing errors and improving overall care. This not only ensures consistent quality of care but also fosters a supportive and collaborative work environment in CH, which benefits both staff and residents (32).

Knowledge management and recognizing intellectual capital have been big topics in business theory for a long time. We are curious about how these ideas apply to CH. CHs are important in long-term care, and there is a growing focus on providing good services and being innovative socially because people are living longer. In CH, knowledge is more than just following rules; it includes understanding the entire environment of working and living in a care home. As Davenport and Prusak [(33), p. 5] state:

“Knowledge is a fluid mix of framed experience, values, contextual information, expert insight a grounded intuition that provide an environment and framework for evaluation and incorporating new experiences and information. It originates and is applied in the minds of knowers. In organizations, it often becomes embedded not only in documents or repositories but also in organizational routines, processes, practices, and norms.”

Knowledge management in CH is essential to ensuring quality and consistent care for residents. The choice of an appropriate knowledge management strategy brings to its implementation through a process of knowledge management cycle that consists of knowledge acquisition, sharing, development, preservation, and application (34). Effective knowledge management enables staff to stay updated, learn from experiences, and continually enhance the quality of care. However, our primary objective is the management of tacit knowledge to improve residents’ living conditions and to create a more stimulating work environment for staff. This, in turn, ensures that the values of a CH are upheld in daily routines (30). Are we aware of different forms of knowledge that are transferred and create new knowledge in CH? CH managers are faced with the extremely difficult task of providing high-quality person-centered care with the aim of ensuring a dignified life and the well-being of residents. The challenge they face derives from two reasons: the increase in the number of older adults exceeds the available capacity of CH and the lack of interest in working in CH leads to a lack of staff (1). Effective knowledge management is essential to innovation. Managers who understand intellectual capital can develop strategies for capturing, sharing, and applying knowledge within organizations to foster innovation (9). The current situation in CHs feels more like crisis management than knowledge management focused on creating new or improved services for residents and the local community.

First, we must identify the knowers. Are they only members of staff, or are they also residents of CH and their relatives? The knowers in CHs are not only caregivers and health professionals, but also residents and their relatives with their experiences and their knowledge. Therefore, when determining tacit knowledge, the importance of the tacit knowledge of users – who are primarily residents of CCIs, but also their relatives who may have been in the role of caregivers for years – should not be overlooked or neglected. The transfer of tacit and explicit knowledge can be influenced by various factors (possible barriers), such as:

1. Staff turnover and training: high staff turnover in CHs can hinder knowledge transfer as new staff may not have the necessary training and experience to implement new practices or interventions effectively.
2. Limited resources: CHs often face resource constraints, including limited funding, staff shortages and lack of time, which can hinder the adoption of new knowledge or practices.
3. Resistance to change: Staff or management resistance to change can be a significant barrier to knowledge transfer. Employees may be reluctant to adopt new practices due to fear of increased workload, uncertainty or adherence to existing routines (29).
4. Communication challenges: Inadequate communication channels or methods in CHs can hinder knowledge sharing between staff. Poor communication can lead to misunderstandings, errors and inefficiencies in implementing new practices.
5. Hierarchy and power dynamics: Hierarchical structures in CHs can hinder knowledge sharing between different levels of staff. Power imbalances can prevent lower-level employees from speaking up or contributing ideas, thus hindering knowledge transfer (31).
6. Regulatory and quality management system: Regulatory requirements or policies within CHs do not always align with or support the implementation of new knowledge or practices, creating barriers to change, or the quality management and improvement process does not promote knowledge transfer.

### 1.3 Aim and research question

The aim of this study was to explore the perceptions of knowledge transfer in CH among staff members, residents, and relatives. This study sought to answer the following research question:

- What facilitators and barriers to implementing knowledge transfer exist in a CH?

The results of this study could be used to inform the development of a knowledge transfer intervention in CH in aging society.

## 2 Materials and methods

This study systematizes existing literature on the knowledge ecosystem in CH and puts on the map of knowledge in CH also residents and their relatives. Specifically, our analysis highlights identification of knowledge of residents and relatives as experts from experience and knowledge transfer in CH. Second, the CH can be seen as a gerontological center in the local community.

Our paper proposes an interpretive framework for knowledge management activities as an important role of leadership.

In this qualitative longitudinal study, three focus group interviews were conducted in a CH in Slovenia with staff and residents and their relatives to explore the experiences of knowledge transfer. Two focus groups were conducted in 2020. The first one included three staff members, four residents, and two relatives (*n* = 9). The second focus group was targeted at staff members in the same CH (*n* = 8). The third focus group was conducted in 1 and included four staff members, five residents, and three relatives (*n* = 12). The staff members who cooperated in the focus groups were caregivers, nurses, occupational therapists, physiotherapists, and a director of CH. All participants gave their informed consent to participate in this study.

### 2.1 Data collection

Project group from the Faculty of Social Work and the Faculty of Social Sciences of the University of Ljubljana conducted the interviews remotely using Microsoft Teams; five students were responsible for guiding the discussion and five took notes and made transcripts under the guidance of one of the authors (AK). The guidance of the students for the interview and the selection of interview themes was conducted by the same researcher with qualitative research experience who conducted the interview in 1. A semi-structured interview sheet guided the discussion (Table 1).

**Table 1:** Interview question guide.

Interview question guide	Examples of questions
Transfer of knowledge in the CH	How is knowledge transferred in the CH between staff (within the unit and between units); between staff and residents (how, in which areas, and with what goal and purpose); and between staff, residents, and relatives (how, in which areas, and with what goal and purpose)? What opportunities, facilitators, and obstacles do you face in digitizing services, informing relatives, and transferring knowledge? In which areas do you already use ICT? What are the mutual relations with colleagues, residents, and relatives? How is it done the exchange of information and wishes?
Knowledge transfer from the CH to the community	What is the role of the CH in the community in which it operates? How does the CH relate to the community (individuals, groups, societies, and organizations)? What are the plans for connecting the CH with the community in the future?

To begin, the interviewer briefly presented the theme of the conversation to stimulate discussion. The participants were then asked to discuss knowledge transfer. The questions were open-ended, and the interviewer encouraged participants to freely develop the discussion and kept the conversation on-topic. The interviews lasted about 2 hours and were recorded and transcribed verbatim.

The research adopted a qualitative approach in which various data sources and data collection techniques were used to validate the findings through a triangulation process. The information was organized into a database in which the chain of evidence was maintained for each focus group (35).

Data from all focus group interviews were analyzed using content analysis with inductive category development (36). First, all the data were read repeatedly to obtain a sense of the whole. Second, transcripts were read word-by-word while highlighting the text describing knowledge transfer between individuals, from individuals to groups, between groups, from groups to organizations, and from organizations to the community. These highlighted meaning units included words, sentences, or paragraphs that answered the research question. These units were then sorted into sub-categories based on how they were related and linked. Finally, the main categories were formed.

## 3 Results

Each researcher individually coded and categorized data from the focus groups to allow the triangulation of the findings. Data from the focus groups was then coded by one researcher and were reviewed by coauthor. As a result of the analysis, the following four categories were derived to describe the knowledge transfer:

1. Facilitators and barriers related to knowledge transfer between staff.
2. Facilitators and barriers related to knowledge transfer between staff and residents.
3. Facilitators and barriers related to knowledge transfer between staff and relatives.
4. Facilitators and barriers related to knowledge transfer between the CH and the community.

The subcategories included in these main categories are listed in Table 2.

**Table 2:** Main categories and sub-categories related to facilitators (+) and barriers (−) identified in the focus groups.

Main categories of knowledge transfer in CH	Sub-categories of knowledge transfer in CH
Facilitators and barriers related to knowledge transfer between staff	+ A psychologically safe work environment enables**open communication** + Mentoring, an experienced and a novice nurse/caregiver working together + Comprehensive induction for new employees + Possibilities for further education and sharing expertise with colleagues + Working in a multidisciplinary team and having a positive attitude toward it + Leadership that encourages knowledge transfer + Staff seminars and workshops where knowledge and skills can be shared with colleagues + Electronic resident record system + Daily reports about residents + Regular team meetings + Face-to-face and electronic communication + Agreed-upon and documented work processes and practices -**Lack of staff and time** - Excessive workload and the resulting difficulty in finding time to mentor new employees/students - Reluctance to learn how to use digital devices/platforms or uncertainty using them
Facilitators and barriers related to knowledge transfer between staff and residents	+**Open communication** + Person centered care + Gathering information about the preferences, habits, and life history of a new resident from the resident and their loved ones + Attitude toward older adults; equal care, respect, kindness, and trust + A communal and reciprocal CH culture + An individual care plan for each resident + Social contact between staff and residents + Planned, joint activity sessions for residents and staff + Collaborative projects + Utilizing residents’ skills in everyday life to create a feeling of usefulness for them -**Lack of staff and time** - Care work causes staff physical, psychological, and emotional strain - Residents’ modesty and reluctance to be a burden to busy staff - Residents’ poor functional, cognitive, and communicative abilities - Underutilization of residents’ resources and abilities
Facilitators and barriers related to knowledge transfer between staff and relatives	+**Open communication** between the CH staff and relatives, and vice versa + Effective collaboration with relatives during a resident’s transition phase + Utilizing the expertise of a resident’s family members/relatives in the life of the CH + Having a website and Facebook account to enhance communication -**Lack of staff and time** - Stereotypical attitudes of relatives toward CH - Exceptional circumstances, such as Covid-19 restrictions on visiting relatives
Facilitators and barriers related to knowledge transfer between a CH and the community	+**Open communication** + Creating an informative webpage for a CH + Creating instructional and support materials for family caregivers and home care + Collaboration with other organizations in the community + Open events/activities for everyone at a CH + Creating opportunities for interactions between different generations + Changing the negative stereotypes often associated with older adults - Negative attitudes toward aging and living in CH - The media’s interest in negative issues and events

### 3.1 Facilitators and barriers related to knowledge transfer between staff

Clear communication channels, open interaction, and multidisciplinary teamwork effectively facilitate the transfer of knowledge within the CH. The CH professionals described that formal knowledge transfer takes place through the electronic resident information system, daily reports during shift changes, and weekly team meetings. Exchanging informal information either in person or through notes and emails was also perceived as facilitating knowledge transfer.

“This report book is excellent because it consolidates all the information and details for each resident in one place. It also allows us to convey general announcements for the entire department, which we place at the top. Additionally, we can specify the duration for which the general notice will appear on the page. So, it truly serves as a highly functional tool” (Staff member).

Multiprofessional collaboration and teamwork were also considered highly important. The CH professionals believe that a permanent team, in which everyone gets to know each other and the residents well, works best in problem solving. Working as a team enables learning from each other and the transfer of tacit knowledge.

“We also include occupational therapists and physical therapists to collaborate with nurses. This ensures that everyone learns proper techniques for moving residents and handling wheelchairs and bedrests and regularly updates these skills. We also introduce them to various tools and equipment. I consistently encourage them to seek assistance if they encounter any issues or challenging situations. I emphasize the use of elevators. I always stress that we work as a team, so we can collectively and efficiently address any problems that may arise” (Manager).

The CH staff emphasized the importance of comprehensive onboarding and mentoring for new personnel. A new or novice employee is paired with an experienced professional, which promotes familiarization with work processes and procedures and facilitates various forms of knowledge transfer. However, mentoring was also perceived as challenging due to heavy workloads and insufficient time.

“Each new employee is assigned a mentor, and knowledge is transferred accordingly. After the mentorship period, which typically lasts for about a month, the new employees are paired with experienced staff members, and they collaborate as part of the team” (Manager).

“Everybody appreciates the support of senior staff members, making them an integral part of the team. Even when someone stands out, the team quickly integrates them. There does not appear to be any friction stemming from age differences” (Manager).

“I would say that being a mentor can be quite challenging at times. However, when you take on the role of a mentor for a new staff member, it becomes your responsibility to effectively transfer your knowledge to them while carrying out your duties. Our work is often like that; we frequently find ourselves lacking the time to talk to them about a case, which could potentially enrich their knowledge as well” (Staff member).

The CH staff emphasized the importance of a psychologically safe work environment and a positive atmosphere for effective information transfer. The interviewees described a prevailing culture of relationships that embodies trust and connection. This trust is nurtured through consistent positive interactions, resulting in a cohesive team that operates efficiently across all organizational tiers. They described that this phenomenon becomes evident in daily routines, during the integration of new members into the CH and when addressing significant challenges. The effectiveness of teamwork is intrinsically tied to the emotional intelligence of its members. Notably, this pertains to collaborative dynamics, where the quality of the relationship itself holds significant value. Every staff member contributes to fostering a positive environment in the CH, thereby fostering mutual trust. Leadership plays a pivotal role in establishing such an atmosphere and fostering a cohesive work environment.

“This trust between us is very important. Each person brings something new, a sense of freshness” (Staff member).

“We eagerly anticipate the time we spend together. If we ever run out of ideas, we brainstorm together, and if nothing else, we find someone to bounce ideas off. We process things in our own unique way, you know?” (Staff member).

Staff shared that there are some among them who are hesitant about using digital communication tools and technologies. This can impede the transfer of information, prompting them to perceive a meticulous process of adopting digital tools as crucial. This process, they believe, would enhance everyone’s competence and alleviate any apprehensions.

“It’s evident that some employees aren’t particularly enthusiastic about adopting ICT solutions. Personally, I believe that this new technology might not be well received by older employees, as many of us are more comfortable with traditional tools like a simple notepad and pen. So, integrating these new systems alongside our current practices could pose some challenges” (Staff member).

### 3.2 Facilitators and barriers related to knowledge transfer between staff and residents

A communal organizational culture, open communication and a positive atmosphere were also recognized to be crucial for the transfer of information between staff and residents. The residents felt that they received assistance and the best possible care from the staff, despite the staff shortage. They also felt comfortable suggesting activities that they personally preferred to the staff. During these activities, both parties learn from each other.

“The facility has successfully fostered an atmosphere that fosters a sense of connection between residents and employees. They are not isolated from the community or their families. As one resident mentioned, … I feel like I’m part of one extended family here” (Staff member).

“Every resident has his/her own preferences when it comes to activities and food. The key with food is to provide them with what they crave. To understand each individual and what you can offer them” (Staff member).

“We also occasionally make suggestions to the employees. There have even been times when I have felt that there could be a slight improvement in the way things were being cooked, and I made suggestions. It led to significant improvements” (Resident).

The staff explained that they aim to engage in various activities with the residents, such as baking, crafting, and playing games. They also gather for a daily shared coffee moment. They also undertake joint projects, such as creating videos, which helps in transferring knowledge beyond the CH.

“We make a conscious effort to utilize the knowledge possessed by our residents. For instance, we frequently engage in baking and cooking using their recipes. They also teach us various skills like knitting and silk painting. The residents have a wealth of knowledge to share. One old lady, for instance, possesses a great deal of knowledge. With her background working in a kindergarten, she gravitates toward creative workshops and offering general assistance. She still desires to contribute and finds the most joy in recounting her experiences of helping others. We understand the significance of feeling useful” (Staff member).

The CH professionals’ discourse highlights a genuine desire and interest in meeting the residents as they are. The staff gather information about each resident’s preferences, habits, and life journeys from the residents and their loved ones. They shared that it is important to create an individual care plan for each resident. In addition to ensuring quality care, individual plans promote knowledge transfer on multiple levels.

“We are consistently proactive in engaging with our residents’ families to understand the habits and preferences of their loved ones, allowing us to build a stronger connection with each resident. Given the busyness at reception and the volume of information, we provide a welcome folder that includes all necessary contact numbers and essential information. Additionally, we encourage the family members to fill out a life story for the new resident, although there are instances where they may not return this form. However, in most cases, they do. Our goal is to ensure that the individual habits of each resident are well documented and communicated to the in-house care provider, who then shares this information with the rest of the department. Typically, we collaborate with the families to compile the life story, especially considering that our residents often have complex health conditions” (Staff member).

According to the participants, the barriers to the transfer of knowledge between residents and staff include the modesty of many residents, leading to them not sharing information with the staff, as they do not wish to burden them further. It was evident from the staff that care can become physically, mentally, and emotionally overwhelming, and in such cases, interacting with residents may become more challenging. The relatives raised concerns that not all of the older adult’s strengths and skills are utilized in the CH environment. The fact that residents often have poorer functional abilities upon transitioning to the CH can pose its own challenges for knowledge exchange between the staff and residents.

“I’m very modest. I do not usually share my habits with the staff because every resident has their unique habits. With the limited number of employees, it’s challenging to discuss personal habits. Sometimes, I simply tolerate minor inconveniences, but if something is genuinely problematic, I do communicate it, and they consistently address the issue” (Resident).

“Because they work so hard, you cannot help but feel sorry for them. However, in my case, I cannot do much to assist myself. I can manage my exercise routine independently, but I struggle to take care of myself.” (Resident).

“When we were constructing the flower bed using wooden logs, he assisted in flattening the logs. He was also skilled at basket weaving, but now he faces difficulties with his fingers and can no longer engage in basket weaving. Otherwise, he would have taught us that skill as well” (Staff member).

### 3.3 Facilitators and barriers related to knowledge transfer between staff and relatives

The transition phase of a new resident is a critical time for the transfer of information and collaboration with family members. Relatives provide crucial information about residents’ histories, abilities, and preferences that can be utilized in crafting an individual care plan.

“When it comes to communicating with relatives, it works like this: When residents are admitted to the facility, we are legally obligated to create an individual care plan for each of them. Subsequently, we share this plan with the residents’ families and engage in discussions with them after a resident has settled in. Naturally, we initially seek a residents’ consent if they have not already provided it. Fortunately, they usually agree. This step is crucial because it fosters open dialogue. During these discussions, we outline what a resident’s daily activities involve, including their participation in physical and occupational therapy, social interactions, and medical care procedures” (Staff member).

The relatives noted their appreciation of the ease of reaching out to staff, being kept updated about their loved ones’ conditions, and receiving direct information from various professionals as needed. The professionals emphasized the significance of relatives in getting to know residents, noting that relatives shared their expertise, for example, in caregiving procedures related to their loved one’s specific conditions. The professionals mentioned being able to leverage the expertise of residents’ relatives in various tasks, such as passing on information about the way of care in the home environment, about the factors that affect the well-being of their parents, as well as involvement in the renovation of the home. The staff members share their expertise with relatives, for instance, when assisting residents in safe transfers. The staff mentioned the importance of enabling interaction and accessing information through multiple channels, including face-to-face communication with professionals at the CH, over the phone, through virtual connections, and through information on the CH’s website and Facebook page.

“I prefer talking to a nurse rather than visiting a website or Facebook, but I’m also glad to receive calls from the doctor’s office whenever there’s a change in my love ones’s health; they keep me informed. I believe the media does not recognize the hard work the employees are putting in, and they aren’t appreciated enough. However, in the event of an emergency or any negative occurrence, reporters would be flocking to the door” (Relative).

“At times, we also organize lectures given by relatives. For instance, one relative provided a video on a USB stick demonstrating how to change a pump (for pain relief), while another conducted a presentation for the staff on how to care for her mother, as her illness was unfamiliar to us. She described how she managed her mother’s condition at home. Relatives also occasionally share interesting topics, such as lectures on healthy living, and various general knowledge talks. These discussions cover a wide range of subjects. Furthermore, relatives sometimes showcase their talents by singing, playing musical instruments, and even putting on concerts for the residents” (Manager).

### 3.4 Facilitators and barriers related to knowledge transfer between the CH and the community

The staff pointed out that prior to the pandemic, interactions with the surrounding community were more frequent and included workshops, concerts, and events open to outsiders. These activities have not yet fully returned to their previous state, and currently, there is insufficient staff to facilitate them.

The staff felt that collaboration with relatives and professionals from outside the CH became more difficult during the Covid-19 period, leading to the adoption of alternative methods, such as remote communication. These methods did not work for all residents, for instance, due to sensory issues or because residents preferred face-to-face interactions. Not all activities have returned to their pre-pandemic levels, and the professionals felt that there could be more collaboration with external parties.

The CH instructional videos and materials are freely accessible on their website. Their purpose is to provide information about aging, reduce associated stereotypes, and support activities, such as caregiving for family members. For the staff, it is important to reduce the stigma associated with aging and living in a CH by sharing information with other organizations, such as schools.

“During the Covid-19 pandemic, we were compelled to resort to alternative methods, and we eagerly anticipated the return of in-person contact. We should strive to incorporate more of these personal interactions” (Staff member).

“We have a monthly schedule of activities that is available online, and outsiders are welcome to join at any time. On special occasions, we also extend invitations to the community. Additionally, we offer workshops for external participants, including various cultural and artistic ones. These are valuable and significant endeavors, but unfortunately, we are currently facing challenges in terms of staffing” (Manager).

“For the past few years, I have delivered lectures at schools about aging and its implications and about dementia. This is because students often have grandparents, making it easier for them to comprehend the concept of dementia” (Staff member).

## 4 Discussion

In this study, the ecosystem of knowledge in CH was analyzed, which consists of the knowledge of the staff, the knowledge of the residents and the knowledge of the relatives. Numerous barriers and facilitators to knowledge transfer of knowledge between these three groups were identified. All types of knowledge have an impact on the quality of care and well-being of residents, and the tacit knowledge of stakeholders often represents the pathway to improved practice in CH (30). Understanding how different types of knowledge form the intellectual capital of a CHs is often overlooked as CHs managers’ attention is often focused on measurable and explicit forms of knowledge, such as monitoring responsiveness to the doorbell, recording adverse events, monitoring resident hydration and weight, and assessing functional capacity.

The present study advances our insights into how knowledge transfer can influence human and social capital in CH. We argue that we are unaware of the value of the knowledge possessed by residents and their relatives that could influence service improvement at CH. By creating an organizational culture that promotes knowledge transfer, we build trust and relationships that influence the transfer of tacit knowledge among all those involved in the long-term care process at CH. Given the lack of spare capacity in CH, the increasing proportion of older adults, and the focus on community-based long-term care services, CH can create an intersection of knowledge about older adult care and become a gerontological hub in a community.

**Figure 1:**
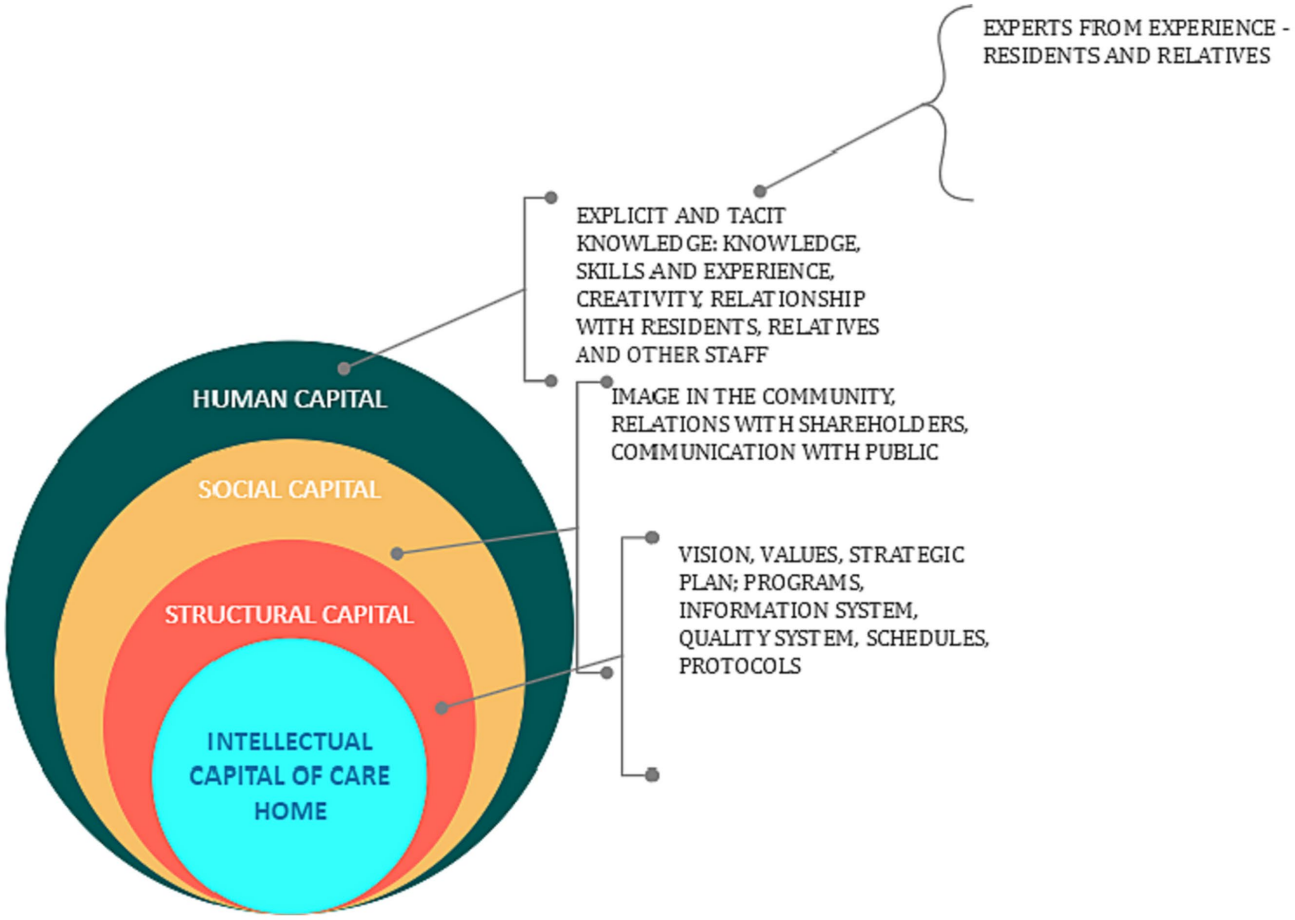
Intellectual capital of care home.

Relatives and residents are experts due to their experiences and should be heard and communicated with in the CH environment (Figure 1). Yet, they are often overlooked and unheard. Experts by experience are individuals or groups who share a common experience of social and health issues. These individuals or groups are classified as peers who have common experiences and can provide various types of support for someone who is new to the experience or is entering recovery (37–38). Service user involvement in the education of health professionals varies from service user academic positions in some institutions to service users contributing to classroom discussions in others (39), but there is generally a lack of service user input in curricula development (40). Research in the area is only beginning to emerge, and much of what has been published focuses on student and health professional perspectives, with little known about service user perspectives (39, 41–43).

### 4.1 Theoretical implications

Knowledge transfer processes between stakeholders are mediated by the interests of the relevant parties (44), so they must be aware of the value that is generated. The results show that they have a high interest in sharing knowledge, but there are barriers that affect the transfer of knowledge in CH.

#### 4.1.1 Facilitators to implement knowledge transfer in care homes between staff, residents, and relatives

Various knowledge transfer practices have been introduced at CH to encourage the transfer of knowledge between generations and to new employees. These include various organizational measures, such as mentoring; working in pairs; consistent education in CH; teamwork; daily meetings during shift changes; and activities to improve communication and create a safe and stimulating work environment (e.g., adhering to the maxim of respectful communication between all stakeholders, daily team meetings, and the use of modern information technology, such as a computer system where all changes and events for each resident are recorded).

The CH in the present study aims to establish person-centered care, which is based on personal biographies and respecting the wishes, needs, and expectations of its residents. As a result, it creates a personal care plan, which is reviewed every 6 months. It has established permanent teams that know the residents well, resulting in a home-like atmosphere in which, in addition to providing care services, they offer activities that residents might do at home, such as a daily coffee ritual, baking pastries, gardening, and other household chores. An important factor in the transfer of knowledge between employees and residents is a relationship based on trust and mutual respect.

If CH include relatives in daily activities and they are regular visitors to CH, a partnership relationship can be created between employees and relatives whereby everyone works hard for the well-being of residents. The partnership relationship is based on immediate and clear communication between employees and relatives. Relatives expect real-time notifications about any changes in their loved one’s health, and they also expect to be heard. After all, they have taken care of their loved one for many years and know what contributes to their well-being.

In this study, we noticed that in an organizational culture that lists trust, respect, and knowledge among its values, a rich ecosystem of knowledge is created, which contributes significantly to the intellectual capital of a CH. From the beginning of this research, we encouraged the creation of a knowledge portal, where the CH collects knowledge about, for example, best practices in care, the rights of older adults, and dementia and distributes it to the local community, thereby functioning as a true gerontological center.

#### 4.1.2 Barriers to the implementation of knowledge transfer in care homes

The study revealed some important barriers to knowledge transfer within care homes. Staff encounters challenges due to a lack of time, exacerbated by excessive workloads and hesitancy in adopting digital tools. Staff shortages and residents’ modesty, cognitive limitations, and underutilized resources further impede effective knowledge exchange. Stereotypical attitudes among relatives, compounded by exceptional circumstances like COVID-19 restrictions, hinder knowledge transfer between staff and family members. Negative community perceptions of aging and care homes, perpetuated by media focus on adverse events, present additional hurdles.

To overcome these barriers, fostering a culture of continuous learning and mentorship, along with addressing staff shortages, is essential. Initiatives promoting digital competency can enhance staff’s comfort with technology. Encouraging open communication with residents and recognizing their untapped potential can facilitate knowledge transfer. Challenging stereotypes among relatives through educational programs and adapting to exceptional circumstances are crucial. Finally, community outreach efforts emphasizing positive aspects of aging and care homes can reshape public perceptions, fostering more effective knowledge transfer from care homes to the broader community.

### 4.2 Limitations and future research

Some methodological considerations had to be considered in this study. Data was collected in two different phases, and different interviewers conducted the interviews, which may have resulted in variations in the interview process. However, the same experienced researcher, who conducted the latter interview, was responsible for the planning of the interviews and the guidance of the interviewers in the first round as well. Although efforts were made to organize the interviews so that the CH staff and residents, as well as their relatives, could participate and have all their perspectives represented in the data, the staff’s voice prevailed simply because they spoke more during the interviews. Studying other CH may have yielded different results. Due to the limited sample size, the results of our study cannot be generalized to all CH, even in Slovenia. However, our findings shed light on the means, facilitators, and barriers to knowledge transfer that are likely to recur in a broader context, so they can be applied to a wide range of CH. The study’s confirmability was strengthened by how we, as research partners with different perspectives and various professional backgrounds, worked on the data and obtained consistent results from the analysis.

Future work could explore how knowledge transfer in the context of CH could be facilitated and intellectual capital strengthened. It would be interesting to study, for example, how leadership style affects knowledge transfer processes within CH. Future studies could also examine the factors related to the relationship between intellectual capital, quality of care, and the well-being of residents in CH.

## 5 Conclusion

This study revealed important facilitators and barriers that promote or hinder effective knowledge transfer in CH. Taking into consideration the facilitators and barriers identified in this study helps to deepen the understanding of how intellectual capital is formed within an organization. There is limited research into human capital in the context of intellectual capital of the CH. Our study extends the discussion on the barriers and facilitators of knowledge transfer within the CH environment, which is valuable in long-lived societies where the need for care and understanding of aging is increasing. To ensure sufficient skilled staff in CH, it is crucial to establish an ecosystem that fosters efficient knowledge transfer, safeguards the well-being of staff and residents, and supports the participation of the surrounding community. There are more and more older adults who cannot afford to live in a CH, despite the need for medical care and social care, so it is important that CH establish the role of a gerontological center in the community.

## Data availability statement

The original contributions presented in the study are included in the article/supplementary material, further queries can be directed to the corresponding author(s).

## Ethics statement

The studies involving human participants were reviewed and approved by CS DEOS Zimzelen Topolšica Strokovni svet. The studies were conducted in accordance with the local legislation and institutional requirements. Written informed consent for participation in this study was provided by the participants’ legal guardians/next of kin.

## Author contributions

AK: Conceptualization, Data curation, Funding acquisition, Investigation, Methodology, Project administration, Supervision, Validation, Visualization, Writing – original draft. KT: Formal analysis, Methodology, Supervision, Validation, Visualization, Writing – original draft, Writing – review & editing.
